# Myocardial ischemia in the absence of epicardial coronary artery disease in Friedreich's ataxia

**DOI:** 10.1186/1532-429X-10-15

**Published:** 2008-04-08

**Authors:** Subha V Raman, Jennifer A Dickerson, Roula Al-Dahhak

**Affiliations:** 1 The Ohio State University and Nationwide Children's Hospital, Columbus, OH, USA

## Abstract

We present the first *in vivo *detection of microvascular abnormality in a patient with Friedreich's ataxia (FA) without epicardial coronary artery disease using cardiac magnetic resonance (CMR). The patient had exertional chest pain and dyspnea prompting referral for cardiac evaluation. These symptoms were reproduced during intravenous adenosine infusion, and simultaneous first-pass perfusion imaging showed a significant subendocardial defect; both symptoms and perfusion deficit were absent at rest. Epicardial coronaries were free of disease by invasive angiography; together, these findings support the notion of impaired myocardial perfusion reserve in FA.

## 1. Introduction

This report describes myocardial perfusion reserve abnormality in Freidreich's ataxia (FA), a heritable disorder whose major manifestations are neurological and myocardial disease. In more than 90% of cases, this autosomal recessive condition results from excess of DNA triplet repeats (GAA) in the first intron at the end of exon 1 leading to suppression of FRDA (frataxin) gene expression. Around 5% of patients have a point mutation in the frataxin gene; both forms result in a deficiency of the protein frataxin that is located in the inner mitochondrial membrane[1]. Frataxin deficiency results in mitochondrial iron accumulation in neurons, cardiomyocytes and other cell types. Historically, diagnosis of cardiac involvement in FA has relied on nonspecific electrocardiographic abnormalities and imaging-based detection of ventricular hypertrophy[2], neither of which yields significant insight into mechanisms of disease that would allow development of targeted therapies. We sought to test the utility of cardiac magnetic resonance (CMR) in identifying microvascular abnormalities in FA, a previously unexplored target for cardiotherapeutic intervention in this disorder.

## 2. Case presentation

**Figure 1:**
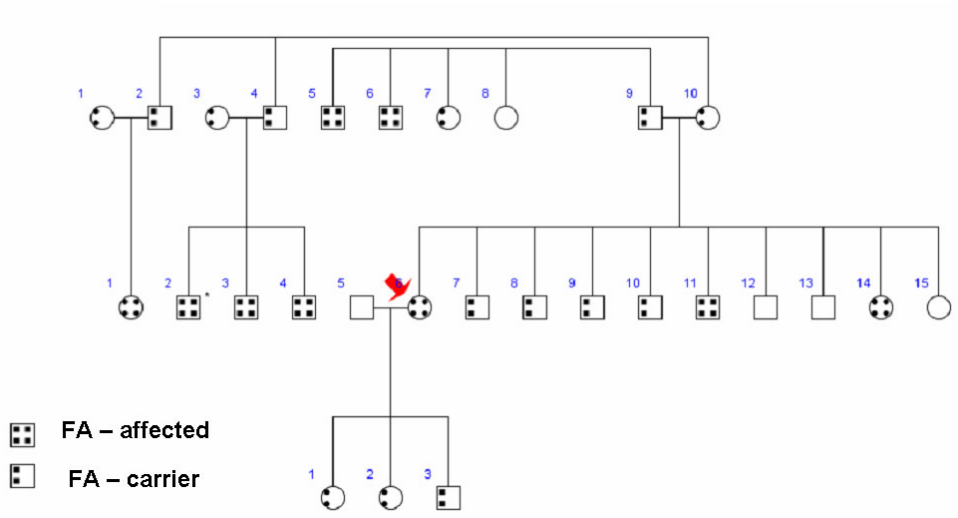
Pedigree shows the extent of Friedreich's ataxia in the family of the patient (arrow).

A 26 year-old nonsmoking Middle Eastern woman presented for evaluation of exertional chest pain and shortness of breath. The past medical history was notable for genotype-proven Friedreich's ataxia (FA) diagnosed at age 19 that had produced ambulatory limitation with frequent falls due to her ataxia. Genotyping showed 846GAA repeats (normal ≤ 33) consistent with FA. Family history revealed extensive affected members and carriers (Figure 1). Further, a nephew with FA had died suddenly at age 21 of presumed cardiac etiology. There was no history of diabetes or hypertension, though serologic examination revealed elevated low-density lipoprotein level (199 mg/dL); serum triglyceride level was within normal limits (57 mg/dL). Cardiovascular physical examination was unremarkable. To evaluate for possible cardiomyopathy as well as myocardial ischemia, she was referred for cardiac magnetic resonance (CMR) examination with adenosine stress perfusion imaging.

**Figure 2:**
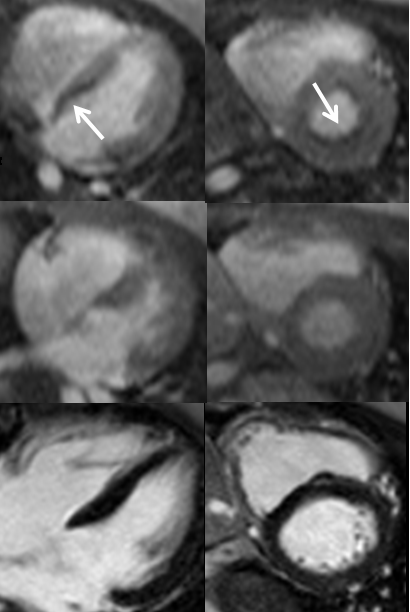
**Stress perfusion (top), resting perfusion (middle) and late post-gadolinium imaging shows a significant subendocardial perfusion abnormality, most prominent along the basal inferoseptum as seen in the horizontal long axis (left) and basal short axis (right) planes.** Corresponding LGE images show no hyperenhancement in the region of perfusion abnormalities consistent with absence of infarct scar or fibrosis.

CMR was completed on a 1.5 Tesla clinical scanner (Magnetom Avanto, Siemens Medical Solutions, Erlangen, Germany) with a 12-channel phased array coil. Resting steady-state free precession cine imaging showed normal left ventricular (LV) size and systolic function, with LV end-systolic volume 21 cc/m^2 ^and LV ejection fraction 62%. Pre-contrast multiecho gradient echo imaging of the heart revealed normal myocardial T2* of 35 ms. With intravenous infusion of 140 mcg/kg/min adenosine, the patient developed severe chest pain and dyspnea that were similar to the exertional symptoms she had been experiencing. These were accompanied by perfusion abnormalities on first-pass T1-weighted imaging using a echo-planar sequence with 0.075 mmol/kg intravenous gadolinium-based contrast (Figure 2). Both symptoms and subendocardial perfusion abnormalities resolved with termination of adenosine infusion. Late post-gadolinium acquisitions showed no myocardial hyperenhancement, consistent with absence of myocardial infarct scar or infiltrate (Figure 2).

**Figure 3:**
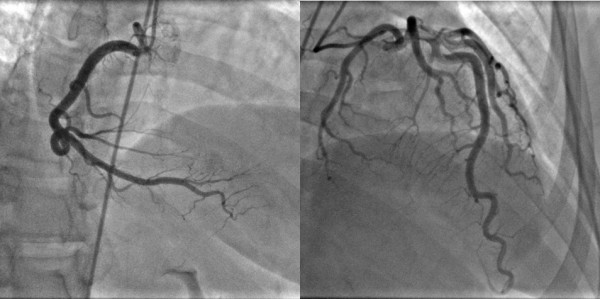
Invasive coronary angiography shows no disease in the epicardial coronary arteries with right coronary artery dominance.

Based on the reproduction of chest pain with adenosine stress and accompanying perfusion abnormality, she was referred for invasive coronary angiography. This showed angiographically normal epicardial coronary arteries (Figure 3) and normal left ventricular end-diastolic filling pressures.

## 3. Discussion

In summary, we identified impaired microcirculatory reserve in FA using CMR that occurred in the absence of epicardial coronary artery disease. Friedreich's ataxia is a neurodegenerative condition frequently accompanied by cardiomyopathy due to a mutation in the gene frataxin, resulting in intra-mitochondrial accumulation of iron. The predilection for neuromuscular and myocardial involvement in FA reflects the high energy demands of both systems.

Prior magnetic resonance-based evaluation of FA cardiomyopathy has focused on cardiac magnetic resonance spectroscopy [3] or quantification of left ventricular mass[4]; to date, CMR perfusion findings have not been reported. The absence of significant iron overload using T2* imaging is not unexpected, given the localization of iron particles in the mitochondria as opposed to the gross iron aggregates that occur in conditions such as hemochromatosis and the transfusion-related secondary iron overload of thalassemia and can produce T2*-shortening. The abnormal myocardial perfusion reserve demonstrated in this young FA patient occurred in the setting of normal epicardial coronary arteries and absence of metabolic syndrome or other known cause of microvascular disease. The only prior investigation of myocardial perfusion was done by Gregory *et al*. using positron emission tomography; their work suggested that compensation for impaired energetics in FA occurs by increasing myocardial oxygen consumption rather than myocardial blood flow, though this study was conducted under resting conditions only[5].

The detection of subendocardial perfusion abnormality afforded by adenosine CMR is similar to that described in other conditions such as syndrome X and coarctation of the aorta[6,7], though the mechanism for abnormal myocardial perfusion reserve may vary among these disorders. Prospective studies of therapies proven to improve microvascular function, such as nitrates and calcium channel blockers, warrant investigation as methods to attenuate the morbidity and mortality due to cardiomyopathy in FA.

## Competing interests

The author(s) declare that they have no competing interests.
